# Gellan Gum/Alginate Microparticles as Drug Delivery Vehicles: DOE Production Optimization and Drug Delivery

**DOI:** 10.3390/ph16071029

**Published:** 2023-07-19

**Authors:** Henrique Carrêlo, Maria Teresa Cidade, João Paulo Borges, Paula Soares

**Affiliations:** i3N/CENIMAT, Department of Materials Science, NOVA School of Science and Technology (FCT NOVA), Campus de Caparica, 2829-516 Caparica, Portugal; h.carrelo@campus.fct.unl.pt (H.C.); mtc@fct.unl.pt (M.T.C.)

**Keywords:** microparticles, gellan gum, alginate, design of experiments, coaxial airflow

## Abstract

Gellan gum is a biocompatible and easily accessible polysaccharide with excellent properties to produce microparticles as drug delivery systems. However, the production methods often fail in reproducibility, compromising the translational potential of such systems. In this work, the production of gellan gum-based microparticles was optimized using the coaxial air flow method, and an inexpensive and reproducible production method. A design of experiments was used to identify the main parameters that affect microparticle production and optimization, focusing on diameter and dispersibility. Airflow was the most significant factor for both parameters. Pump flow affected the diameter, while the gellan gum/alginate ratio affected dispersibility. Microparticles were revealed to be sensitive to pH with swelling, degradation, and encapsulation efficiency affected by pH. Using methylene blue as a model drug, higher encapsulation, and swelling indexes were obtained at pH 7.4, while a more pronounced release occurred at pH 6.5. Within PBs solutions, the microparticles endured up to two months. The microparticle release profiles were studied using well-known models, showing a Fickian-type release, but with no alteration by pH. The developed microparticles showed promising results as drug-delivery vehicles sensitive to pH.

## 1. Introduction

Polymeric microparticles consist of small particles made from a polymeric structure, which range from 1 to 1000 μm [1,2]. These can accommodate drugs/bioactive agents within their structures and withhold their release, thus, having a more prolonged release mechanism compared to oral or systemic administration [2]. There is also the possibility to accommodate cells within their structure, and hence, they can also be a useful system for tissue engineering [3]. Thus, microparticles offer therapeutic and technological advantages, since they could be used as drug delivery systems and also as scaffolds [2].

Microparticles can be divided into two main types of classes, regarding structure: microspheres and microcapsules. The former has a continuous structure within and can have cargo dispersed homogeneously within and the latter consists in a core (where the cargo is) and an outer shell [2]. Within this study, microspheres will be developed (which will be interchangeably referred to with the term microparticles along the text).

There are different production methods available for microparticle production. Emulsion-based techniques consist in water-in-oil (w/o) or oil-in-water (o/w) emulsions, where the drug and polymer are dissolved/dispersed in the appropriate phase [4,5]. The emulsion is then subjected to mechanical shear forces that form a droplet within a matrix solution. This technique has good microparticle size control with high drug loading capacity; however, the use of oils and organic solvents means that after-production washing is a necessary step, due to toxic residues that might be present within the microparticles [5,6,7,8,9]. Another method is the microfluidic technique [10]. In this, a dispersed phase, such as a polymeric solution, is injected through a microscopic channel into a continuous, immiscible phase. This technique can create reproducible monodispersed micro- and nanoparticles [11]. However, the production rates can be low, as well as expensive due to the complexity of the equipment, and these systems are very prone to channel blockage, stopping the operation [12].

Extrusion-based methods consist of droplet generation through a nozzle followed by its hardening in a bath. It is a method that has been used in several studies, since it can produce millimetric particles and microparticles with excellent production rates, reproducibility, and low production costs [13,14,15]. The detachment of the drop can be aided by different forces, including vibration [16], electric field, called coaxial electrospray [17,18], or air, denominated the coaxial airflow technique [16,19,20]. The latter is a replicable and inexpensive method that can produce large quantities of particles. It consists of a nozzle that has a coaxial air stream system parallel to it. When a polymeric solution is extruded through the nozzle, the airflow will aid in the detachment of the drop. This drop will fall in a coagulation bath, which promotes the crosslinking of the polymer [16,21]. This inexpensive method is useful to produce large quantities of replicable microparticles. Additionally, the production does not need solvents or oils that might leave toxic residues in the polymeric matrix.

The use of natural polymers to produce microparticles has several advantages and they have been used for biomedical applications due to their biocompatibility, biodegradability, and biomimicking properties [22]. For example, Alginate (Alg) has been used for microsphere production, including the coaxial air flow technique, due to its sensibility with calcium ions that instantaneously form an ionically linked structure [8,16,23]. Gellan gum (GG) is also a polysaccharide that has good biomedical applications due to its biocompatibility, biomimicking, and good mucoadhesive properties [24,25]. It is a polymer that has been used for different biomedical applications, including in microparticle shape. Unlike Alg, GG microsphere production using the coaxial air flow technique has yet to be fully studied or optimized [26]. Due to also being ionically sensitive to divalent cations, GG can also be used in the coaxial air flow technique. In fact, GG microparticles were previously made in simple extrusion techniques, but not with the aid of a coaxial airflow [27]. Thus, the study of an inexpensive method with a large production rate of GG-based microparticles is an interesting topic to be studied.

Due to the lack of studies using the GG with the established coaxial air-flow technique, this article will focus on the development of GG-based microparticles made with the coaxial air-flow method. Alg was introduced to the production to decrease the viscosity of the GG solution, thus having gellan gum: alginate microparticles (GG:Alg). The optimization of the production method was carried out using a design of experiment (DOE). To the knowledge of the authors, no article focused on the optimization of GG-based microparticles using this method. The main goal of this work was to decrease the microparticle’s diameter (size) and to decrease dispersibility related to the diameter. After the production’s optimization, characterization of GG:Alg microparticles for drug release was carried out in acidic and neutral environments. For drug release studies, methylene blue (MB) was chosen, which is a cationic dye that is used as a model drug, but which is also used in medical treatments. This was followed by a characterization of the particles for biomedical purposes.

## 2. Results

### 2.1. Production and Optimization of Particles

**Figure 1:**
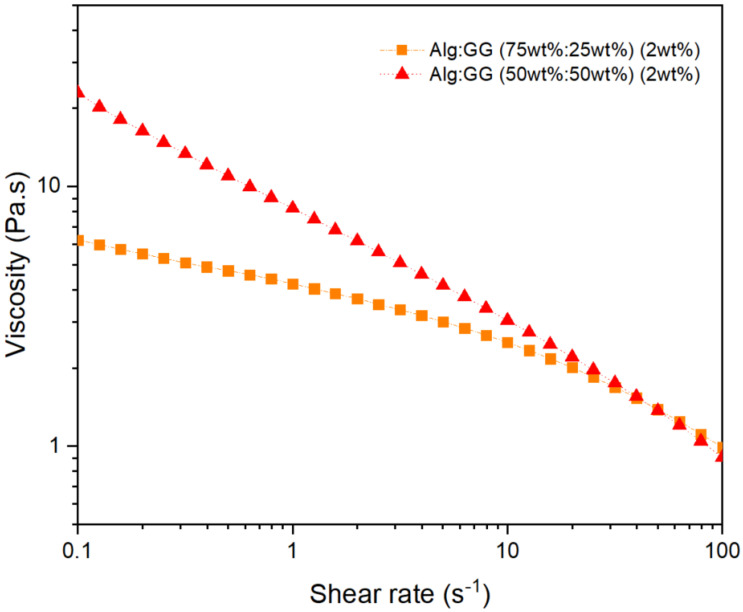
Flow curves (25 °C) of the Alg:GG solutions (2 wt%) with different ratios of GG and Alg.

Microparticle production was performed by coaxial air flow using DOE to optimize particle diameter. Before DOE, preliminary tests were performed to choose the factors and levels. Factors are the parameters chosen to be evaluated in the DOE and levels are the values of these factors that are going to be evaluated. Gellan gum solutions with 1% *w*/*v* clogged the coaxial airflow system, due to high viscosity, using a needle with an internal diameter of 0.25 mm and an outer diameter of 0.5 mm. Reducing the polymer concentration (<1% *w*/*v*) did not allow immediate particle consolidation in CaCl_2_ solution. Therefore, alginate was added to reduce the solution’s viscosity. Maintaining a GG:Alg ratio of 50:50, four concentrations were tested (1% *w*/*v*; 1.5% *w*/*v*; 2% *w*/*v*, and 2.5% *w*/*v*). With 1% *w*/*v* and 1.5% *w*/*v*, the microparticles collapsed and were extremely deformed after falling into the CaCl_2_ bath. With 2.5% *w*/*v*, the solution clogged the system. Thus, the optimal concentration was 2% *w*/*v*, since there was no clogging, and the microparticles did not collapse. Then, maintaining the polymer concentration at 2.0% *w*/*v*, the ratio of GG:Alg was evaluated. Three ratios (GG:Alg) were chosen: 75%:25%, 50%:50%, and 25%:75%. With 75%:25%, there was clogging of the needle. This was due to the different viscosities of the GG:Alg solutions, as can be seen in Figure 1. In this figure, the solutions were revealed to be shear thinning and the solution with a higher presence of GG had a higher apparent viscosity at lower shear rates. This is due to the higher viscosity of GG [28,29]. With higher shears, the viscosities converged, possibly due to the alignment of the polysaccharide chains with the flow [30].

For DOE, the ratio of GG:Alg (factor A) was chosen with the lower level being 50%:50% and the higher level being 25%:75%, with the latter having a higher concentration of alginate. For factor B, the lower level of the bath-nozzle gap was set at a height of 10 cm and the higher level at 20 cm. Airflow (factor C) limits were 2.5 L/min and 5 L/min (the equipment’s maximum). A limit of 2.5 L/min was chosen, because below this level some clogging at the end of the nozzle occurred. Most probably, below 2.5 L/min, the airflow did not exert sufficient force for the drop detachment. For pump flow (Factor D), the lower level was set as 5 mL/h and the high-level set as 10 mL/h. Below 5 mL/h some clogging of the system occurred, so this level was defined as the minimum. Probably, below 5 mL/h, the applied force of the fluid did not allow the drops to form fast enough and detach in even intervals, leading to the hardening of the solution at the nozzle and promoting the clogging of the system. The chosen needle diameter was the previously mentioned with an internal diameter of 0.25 mm and an outer diameter of 0.5 mm. Another needle with a lower diameter (internal: 0.17 mm; outer: 0.3 mm) was used, but the clogging of the system also occurred using the GG:Alg solutions with different ratios. With lower diameters, higher shear rates are applied to the solutions, accordingly to Poiseuille’s equation [31]. Even though higher shear rates are acquired and GG:Alg solutions are shear thinning, and thus, diminishing the solution’s viscosity (Figure 1), the needles with lower diameters clogged frequently and were not able to be used in this study. Thus, needle diameter was maintained as a constant, with the study with larger diameters as a possible topic to be studied for future studies.

**Figure 2:**
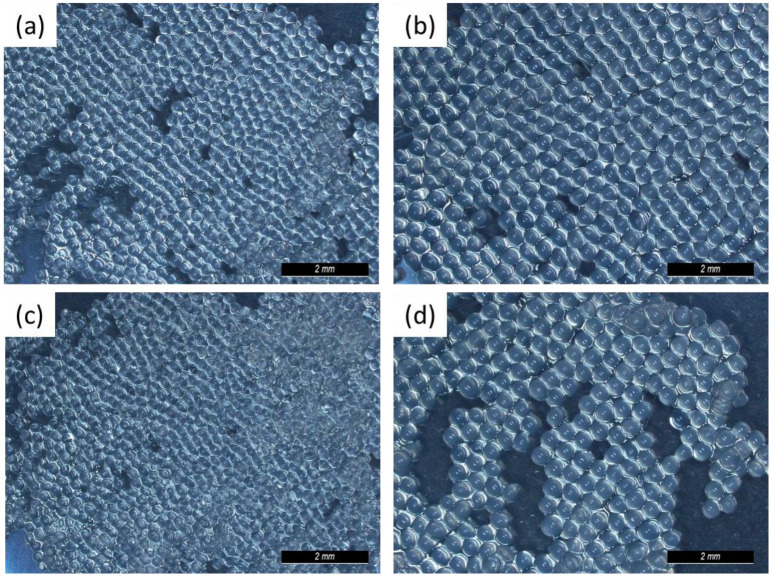
GG:Alg microparticle images from (**a**) run 6, (**b**) run 3, (**c**) run 21, and (**d**) run 10 (see Table 1).

The DOE consisted of 24 runs done in a random sequence (Table 1). All particles had a spherical shape, as can be observed in Figure 2. All results were found to be normally distributed.

#### 2.1.1. Microparticle’s Size

Regarding the microparticle’s size (diameter), only air flow (G) and pump flow (D) were found to be statistically significant, with a *p*-value < 0.05. The GG:Alg ratio (A) and the bath-nozzle gap (B) were not significant, and thus, they were excluded from the ANOVA analysis, as can be observed in Table 2, where only air flow and pump flow were analyzed. Additionally, no significant lack of fit was detected in the ANOVA analysis, as can be observed in Table 2, which was obtained from the Design-Expert Software^®^ (Version 11).

**Figure 3:**
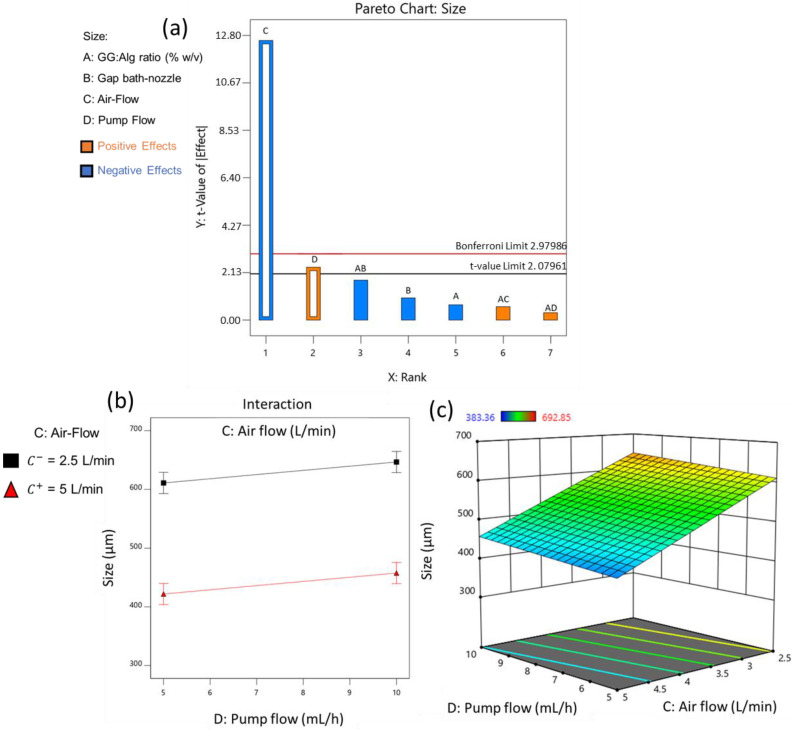
(**a**) Pareto charts with the effects of the different variables on the particle’s diameter (24 runs, at least 100 microparticles measured for each run) (*p*-value < 0.05). Empty bars mean significant factor, while full ones are of non-significant. (**b**) Interaction plots for particle’s diameter between the significant factors the C: air flow and D: pump flow (A: GG:Alg ratio (% *w*/*v*) = 50:50; B: bath-nozzle gap = 15 cm). (**c**) Response surface plot for the particle’s size, with the effect of C: air flow and D: pump flow (A: GG:Alg ratio = 50:50; B: bath-nozzle gap = 15 cm).

Figure 3a represents the Pareto chart for the microparticle’s size, where the effects are ordered by rank (meaning from the one with the highest effect to the lowest) and displays the 5% significance threshold (*t*-value limit) and an additional threshold corrected for multiple testing (Bonferroni limit), given by Design-Expert Software^®^. The Y axis represents the t-value effect of the factors. The t-value relates to the effect of each factor on each response, where the higher the value is, the more significant a factor is to the response; in this case, the size [3]. If a factor has a t-value effect above the t-value limit, it is a significant effect, while if below, it is insignificant [32]. In Figure 3a, for a factor to be considered significant, it only needs to surpass the later limit t-value limit, but if it also passes the Bonferroni limit, there is a higher confidence that the factors are strongly statistically significant. The effects can also be positive and negative. A positive effect means that an increase in the factor will produce a higher response (in this study, larger diameters), while, with a negative effect, an increase in the factor will reduce the response (lower diameters) [32]. On the X axis, the factors are already ranked from the most significant (left) to the least significant (right).

Airflow was the most significant effect, since it had the highest t-value, with a negative effect (Figure 3a). Thus, higher levels of air flow lead to smaller diameters. Pump flow (D) was above the t-value limit but below the Bonferroni limit. Due to its positive effect, higher pump flows, in this case, 10 mL/h, increased the diameter of the particles. The other two factors, the GG:Alg ratio (A) and the bath-nozzle gap (B), had no significant effect on the particle’s size. Figure 3b also confirms that there is no interaction between the statistically significant factors. Figure 3c depicts the effect of air flow and pump flow on size, which shows that the airflow has a far greater impact on the size of the microparticles than pump low.

#### 2.1.2. Dispersibility: COV and SPAN

**Figure 4:**
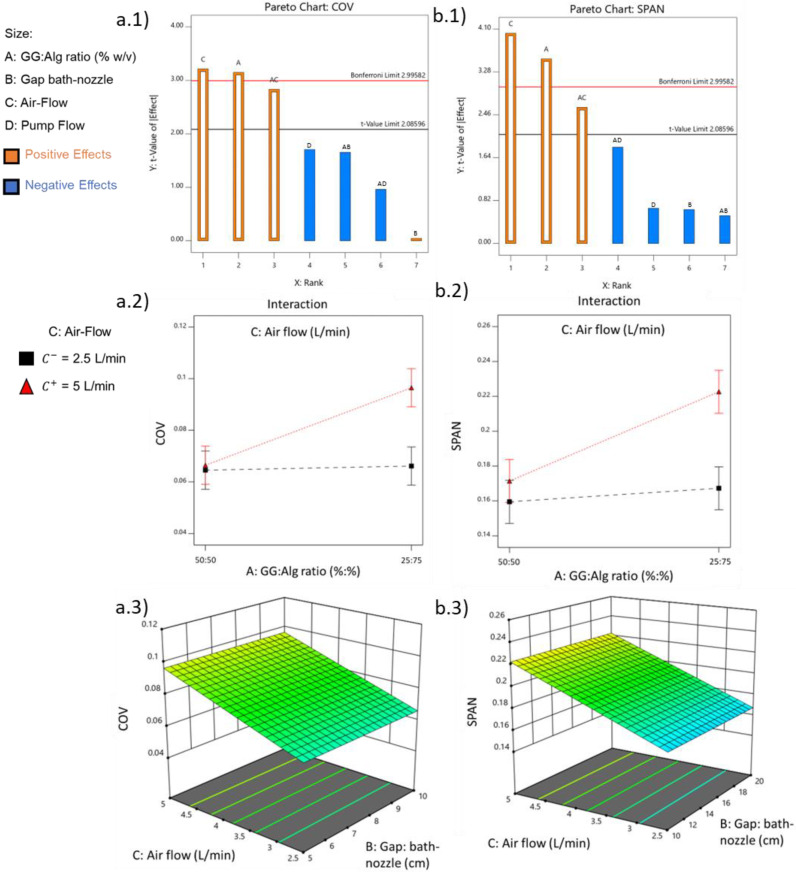
Pareto charts with the effects of the different variables on the particle’s dispersibility (24 runs, at least 100 microparticles measured for each run) (*p*-value < 0.05) (**a.1**) COV, (**b.1**) SPAN. (**a.2**) Interaction plots for particle’s diameter COV between the significant factors the A: GG:Alg ratio and C: air flow (B: bath-nozzle gap = 15 cm; D: pump flow = 5 mL/h); (**b.2**) Interaction plots for particle’s diameter SPAN between the significant factors the A: GG:Alg ratio and C: air flow (B: bath-nozzle gap = 15 cm; D: pump flow = 5 mL/h); (**a.3**) Response surface plot for the particle’s diameter COV, with the effect of C: air flow and D: pump flow (A: GG:Alg ratio = 25:75; B: bath-nozzle gap = 15 cm); (**b.3**) Response surface plot for the particle’s diameter SPAN, with the effect of C: air flow and D: pump flow (A: GG:Alg ratio = 25:75; B: bath-nozzle gap = 15 cm).

Regarding dispersibility, COV varied between 0.4000 and 0.1200 and SPAN between 0.1486 and 0.2496. There was no lack of fit for COV (Table 3) and for SPAN (Table 4). In both responses, the GG:Alg ratio (A) and air flow (C) were statistically significant factors. The interaction between these factors (AC) was also significant. The bath-nozzle gap (B) and pump flow (D) were not significant and were excluded from the analysis. In COV and SPAN, airflow (C) was again the most significant effect, although with a positive effect (Figure 4(a.1,b.1)), meaning that with the higher levels of air flow, higher COV and SPAN values are going to be obtained, and thus, increasing dispersibility. In the GG:Alg ratio, the higher values (associated with higher Alg content and thus lower viscosities) lead to higher COV and SPAN values.

The interaction between air flow and GG:Alg ratio (AC) was also significant, although below the Bonferroni limit in both cases. This interaction was positive, and this was related to the fact that both factors are also positive. Figure 4(a.2,b.2) show the interaction AC for COV and SPAN, respectively. In both cases, the effect of air flow was affected by the GG:Alg ratio. The air flow had more difference between the higher (C^+^ = 5 L/min) and lower level (C^−^ = 2.5 L/min) with a GG:Alg ratio of 25:75 than with 50:50. This is attributed to the differences in viscosity of both ratios (Figure 1). With the GG:Alg ratio of 25:75 the viscosity is lower than with 50:50, so with solutions with lower viscosities, higher air flows will increase the dispersibility of the particle’s size. Figure 4(a.3,b.3)) depict how the COV and SPAN, respectively, are expected to change with air flow and the bath-nozzle gap, where it is observed that the gap nozzle bath does not affect the values.

### 2.2. Drying and Swelling of the Microparticles

**Figure 5:**
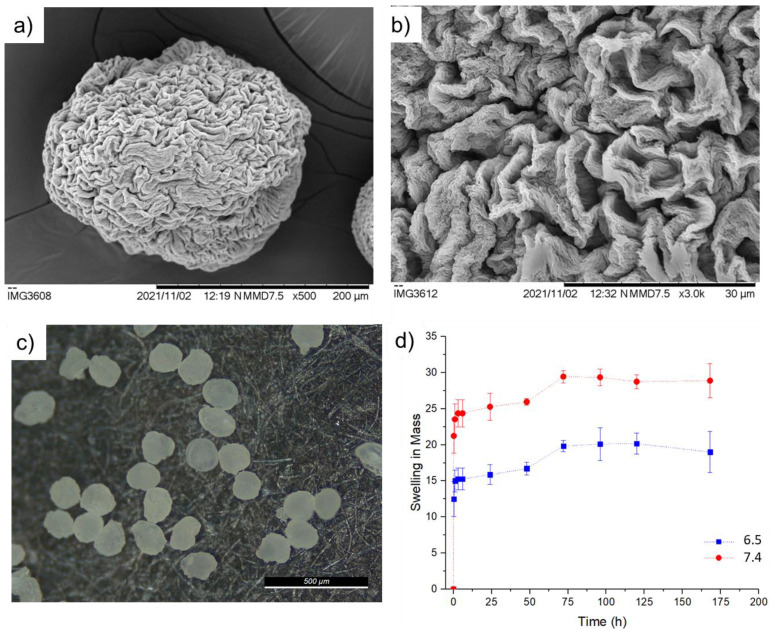
(**a**–**c**) SEM analysis of dried GG:Alg particles. (**d**) Swelling indexes of microparticles.

With the optimized factors to produce particles at their wet stage, the next step was to dry them. Solvent exchange from water to ethanol was performed to prevent the aggregation of particles after drying. The dried particles did not agglomerate and had a round shape. Their surface was characterized by a rough surface (Figure 5a–c). The particles had a diameter between 150 and 220 μm (average of 171 ± 36 μm).

The maximum swelling of the particles reached the maximum at 72 h (Figure 5d). The particles within PBS pH 7.4 swelled to a diameter of 433.55 ± 67.75 μm and within PBS pH 6.5 swelled to a diameter of 398.25 ± 52.37 μm. Particles that were swollen at pH 6.5 conditions presented rougher surfaces when compared to the ones at pH 7.4. With PBS pH 6.5, the surfaces did not “stretch” to their original sizes, leaving some roughness on their surfaces.

### 2.3. Encapsulation Efficiency and Loading Capacity

With swelling studies, it was possible to determine the ideal period in which the particles should be within the PBS solutions with MB. Thus, the particles were left within the PBS solutions with different concentrations of MB for 4 days.

**Figure 6:**
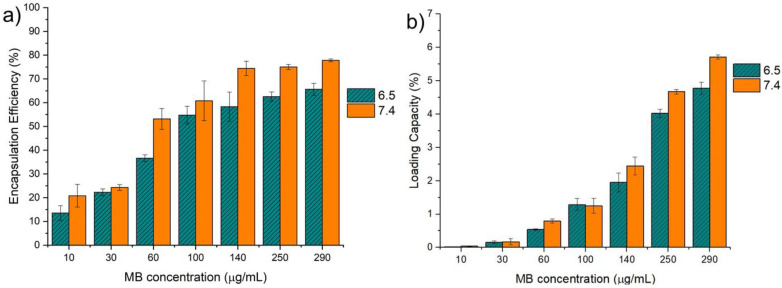
(**a**) Encapsulation efficiency (E.E.%) and (**b**) loading capacity (L.C.) with different concentrations of methylene blue (MB) in PBS solutions with pH of 6.5 and 7.4.

Higher efficiencies and loading capacities were obtained with higher concentrations of MB, as can be observed in Figure 6a,b. With an increase in the initial concentration of MB, the mass transfer resistance against MB diffusion from the solution to the beads decreases. Therefore, the diffusion of the drug is accelerated by an increase in MB concentration in the initial solution [33]. pH also increased the EE% of the microparticles, especially at the higher concentrations of MB, where pH 7.4 had better EE% and LC% than pH 6.5. The best condition of MB encapsulation within the GG:Alg microparticles were obtained with solutions of 290 μg/mL with pH 7.4, with an encapsulation efficiency of 77.83 (±0.56)%. The same was observed with a loading capacity of 5.71 (±0.06)%.

### 2.4. FTIR and TGA

**Figure 7:**
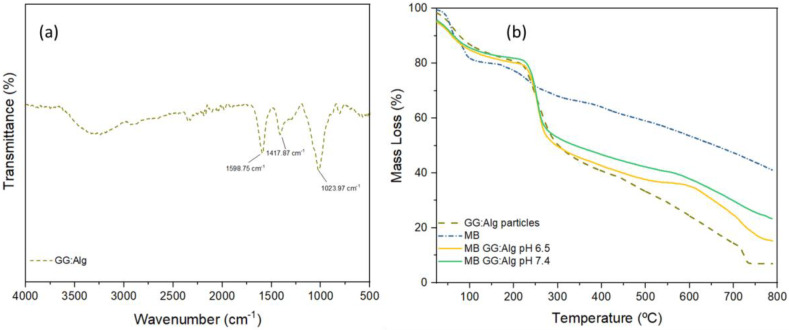
(**a**) FTIR of the GG:Alg microparticles and (**b**) TGA of GG:Alg microparticles, with and without MB, and MB alone.

FTIR analysis of the microparticles without MB was carried out (Figure 7a). Regarding FTIR results of the microparticles, the GG:Alg microparticles had a band around 3400 and 3100 cm^−1^, probably for the -C-H stretching of gellan gum. At 1598 cm^−1^, the peak was attributed to -COO stretching (asymmetric), followed by a -COOH stretching at 1417 cm^−1^ (symmetric) and a C-O-C stretching at 1023 cm^−1^ of alginate [28].

With the optimized loaded microparticles, a TGA was performed on particles with and without MB. All particles and MB alone had a first thermal event that is related to water loss (Figure 7b). MB had a constant mass loss until the final mass of 41.01%. For particles with and without MB, the mass loss at this first event was around 15% and for MB alone around 10%. For all particles, a sharp mass loss occurred between 220 °C and 280 °C. This was caused by the degradation of gellan gum and alginate, previously associated with depolymerization [19,21,34,35]. After this sudden decline, there was a steadier decline in mass until 800 °C. The final residue of the sample without MB was 6.95%. Microparticles with MB retained more mass. Those loaded with PBS pH 6.5 retained 15.24%, and with PBS pH 7.4, they retained 23.34%.

### 2.5. Degradation

**Figure 8:**
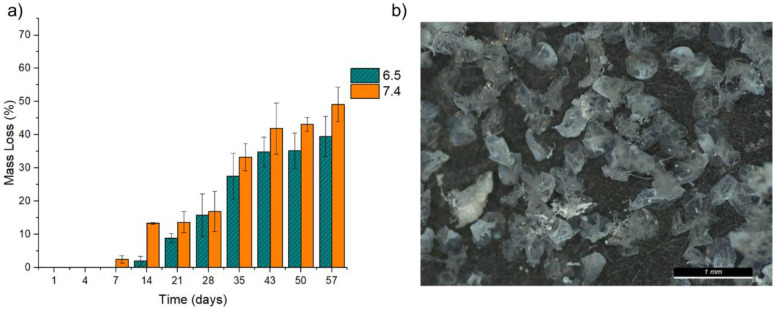
(**a**) Degradation of microparticles within PBS with pH 6.5 and pH 7.4 for 57 days; (**b**) GG:Alg particles after 28 days in PBS.

Degradation only started after 7 days (Figure 8a). Of the two studied pH, pH 7.4 appeared to cause a more pronounced mass loss than pH 6.5. However, one-way ANOVA tests were performed for the last four degradation times: 35 days (F(1,6) = 1.06, *p* = 0.34), 43 days (F(1,6) = 2.73, *p* = 0.15), 50 days (F(1,6) = 4.86, *p* = 0.07), and 57 days (F(1,6) = 6.87, *p* = 0.04). Considering *p* < 0.05, only the last time was statistically significant, with pH 7.4 having higher degradation. Within the 57 days of the assay, the particles lost 49.20 (±5.13) % with pH 7.4 and 39.55 (±5.29)% with pH 6.5 Figure 8a). Although the mass losses averages of pH 7.4 were higher, before 57 days, no significantly significant differences were observed between the mass losses between pH.

After drying, the particles did not exhibit the same morphology that they had before degradation, which may be attributed to the loss of structure (Figure 8b).

### 2.6. In Vitro Drug Release and Mathematical Model Fitting

**Figure 9:**
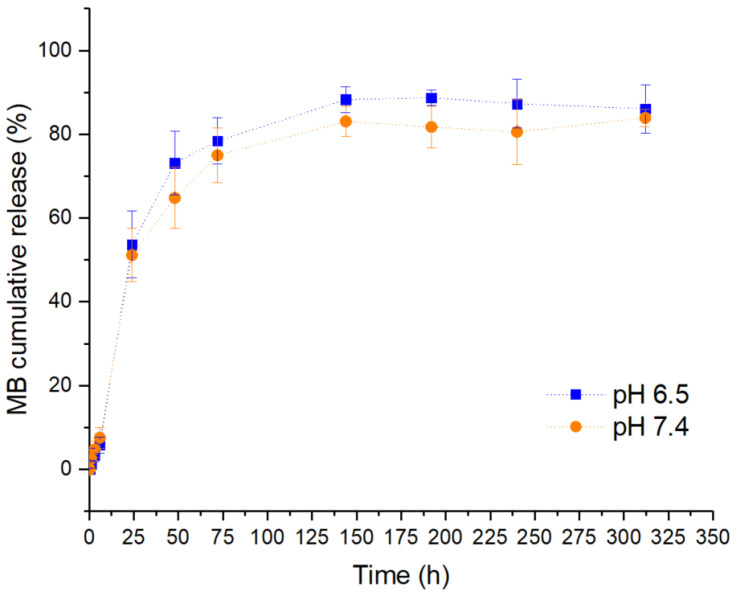
Methylene blue cumulative release from GG:Alg particles in PBS at pH 6.5 and 7.4.

GG:Alg microparticles in PBS, with both pH values reavealing an early burst release pattern [36] (Figure 9). Where a significant part of the MB within the particles was released within the first hours. Within the first day, the particles had released more than half of MB and at 72 h, the particles had released more than 75%. This has been previously reported in alginate particles [37] and in gellan gum particles [38,39]. The burst release might be due to MB molecules on the particle’s surface or within the particles near the surface, where with hydration they are easily released [40]. After the burst release, the release reached a plateau. During the release, there were slightly higher released concentrations of MB with pH 6.5 than with pH 7.4. However, one-way ANOVA tests were performed each time from 24 h onward, to understand if there were statistically significant differences between the release profiles in PBS with pH 6.5 and pH 7.4. The obtained parameters can be observed in Table 5. As can be observed, only at the times of 144 and 192 h were the release profile statistically significant. However, for the rest of the analyzed times, i.e., the majority, there were no differences in the release profiles in PBS with pH 6.5 and pH 7.4. Thus, in general, although the average release is larger in PBS pH 6.5, no statistical significance exists with the release profile in PBS pH 7.4.

For mathematical model fitting, three models were chosen: the Korsmeyer–Peppas (KP), Weibull (W), and Peppas–Sahlin (PS) [25,41,42]. In the Korsmeyer–Peppas model (Equation (4)), $Q_{t}$ is the released drug concentration at each time point (*t*), *k* is a constant of structural and geometric character, and *n* is related to the release mechanism. A modified KP model (KP T_lag_) (Equation (5)) is more suited for swellable systems [43,44], where the lag time before drug release at the beginning of the release ($T_{lag}$) is accounted for [45]. T_lag_ accounts for the time before drug release at the start of the release process [15]. With the Weibull model [42,45] (Equation (6)), *a* is a time scale parameter of the process and *b* is a shape parameter of the release. Peppas–Sahlin model (PS) (Equation (7)) uses two constants that correlate with the contribution of Fickian diffusion (*k_1_*) and the contribution of case II transport (*k*_2_) [46]. The exponent *m* correlates with the KP model and can be associated with the release profile similar to *n* in the KP model [47]. A variation of the PS model (PS *T_lag_*) was also studied (Equation (8)), which, similar to the KP *T_lag_*, model, also accounts for *T_lag_*. From all models, using the variations of KP and SP models with the *Tlag*, better mathematical fits were obtained for the release profiles of MB (Table 6). Both variations of the models revealed that a Fickian release profile dominated the release of MB of the GG:Alg microparticles (*n*, *m* < 0.43). Thus, diffusion is the main driver of the release and not swelling [48]. Additionally in the PS and PS *Tlag* models, the *k*_1_ parameters are greater than *k*_2_, and thus, a higher contribution of the Fickian release was registered. Using the Weibull model, a Fickian release profile was also obtained. However, when compared to the modified KP and PS models, the Weibull model had a reduced R2adj, making a not as good a fit as the others. Regarding *Tlag*, in the KP *Tlag*, the *Tlag* was greater than with pH 6.5 than with pH 7.4. Thus, with pH 6.5 the release started after with pH 7.4; however, with the PS *Tlag* model, the *Tlag* between the pH 6.5 and pH 7.4 were similar.

Table 6 Parameter values and R2adj from the fittings of the mathematical models from the release profiles, from DDSolver. The best fit for each system is in bold.presents the models’ parameters and adjusted R^2^ (R^2^_adj_) given by the DDSolver program.

(1) $$Q_{t}=kt^{n}$$

(2) $$Q_{t}=k{\left(t-T_{lag}\right)}^{n}$$

(3) $$Q_{t}=100\left(1-e^{-tb/a}\right)$$

(4) $$Q_{t}=k_{1}t^{m}+k_{2}t^{2m}$$

(5) $$Q_{t}=k_{1}{\left(t-T_{lag}\right)}^{m}+k_{2}{\left(t-T_{lag}\right)}^{2m}$$

From all models, using the variations of KP and SP models with the *T_lag_*, better mathematical fits were obtained for the release profiles of MB (Table 6). Both variations of the models revealed that a Fickian release profile dominated the release of MB of the GG:Alg microparticles (*n*, *m* < 0.43). Thus, diffusion is the main driver of the release and not swelling [48]. Additionally in the PS and PS *T_lag_* models, the *k*_1_ parameters are greater than *k*_2_, and thus, a higher contribution of the Fickian release was registered. Using the Weibull model, a Fickian release profile was also obtained. However, when compared to the modified KP and PS models, the Weibull model had a reduced R^2^_adj_, making a not as good a fit as the others. Regarding *T_lag_*, in the KP *T_lag_*, the *T_lag_* was greater than with pH 6.5 than with pH 7.4. Thus, with pH 6.5 the release started after with pH 7.4; however, with the PS *T_lag_* model, the *T_lag_* between the pH 6.5 and pH 7.4 were similar.

## 3. Discussion

The particle’s diameter significantly decreased with higher air flows, while it increased with higher pump flows. However, pump flow did not impact the particle’s size as the air flow did. In an atomization process, Chan et al. [49] also found that an increase in volumetric liquid flow rate (equivalent to pump flow in this work) also produced larger particles. Similar effects of the air flow in microparticle’s diameter were also observed in previous works, with higher air flows decreasing the size [20,50]. Nastruzzi et al. [50] also found that height (equivalent to bath-nozzle gap) and flow (equivalent to pump flow) also did not significantly affect the particle’s size. The use of needles with different diameters should also be studied in future works, since the needle affects the shear rate [31]. The needles’ diameter is expected to impact the size of the microparticles. For example, in a coaxial electrospray technique, Anani et al. [51] observed that needles with smaller diameters produce smaller sizes. In this study, smaller needles were experimented; however, clogging occurred. However, other sizes could be experimented.

Higher SPAN and COV values were obtained with higher airflows. Chan et al. [49] found that a lower air-to-liquid mass flow rate ratio resulted in lower particle size distribution. In terms of t-value effects, airflow had a higher effect on SPAN than on COV. The ratio of the GG:Alg (A) had the second-highest effect on dispersibility. Being a positive effect, higher dispersibility occurred at the higher level of 25%:75% (with a higher concentration of alginate and less gellan gum). This was attributed to viscosity. Higher contents of gellan gum increased the viscosity of the solutions, so using lower viscosity solutions increased the dispersibility of the particles.

The main goal was to minimize the size and dispersibility of the particles. For the GG:Alg ratio (A), 50%:50% was selected, since it decreased dispersibility. For factor B, the bath-nozzle gap did not affect the microparticle’s size or dispersibility; therefore, the intermediary level of 15 cm was chosen. Airflow (C) was the most significant factor in all responses. Using higher airflows, smaller diameters were obtained; however, an increase in dispersibility also occurred. On the other hand, airflow had a much higher effect on microparticle size than on COV and SPAN. Thus, this factor had a much higher impact on size than on dispersibility. Thus, the higher level, 5 L/min, was chosen to obtain smaller particles. Regarding pump flow (D), the lower level of 5 mL/h was chosen, since it affected the diameter of the particles with a positive effect and had no effect on dispersibility. The wet microparticles produced in these conditions had a diameter between 400 and 450 μm.

With these results, future works can compare their production of similar microparticles made with the coaxial air flow technique. However, other factors also need to be accounted for.

Higher swelling indexes were obtained with neutral pH. This was attributed to the fact that both polymers are anionic polysaccharides. Carboxylic acid groups of both polymers undergo deprotonation in more basic environments, leading to more negatively charged groups and a decrease in the strength of intermolecular hydrogen bonds [52,53]. The increase in the negativity of the chains leads to repulsive electrostatic charges between chains, thus promoting the spaces between the chains and promoting the penetration of the liquid. Additionally, at lower pH, calcium ions dissociate from the structure, promoting the formation of hydrogen bonds, leading to a more closely packed structure, and preventing higher swellings [54]. These results are in accordance with previous studies that also found that particles with alginate [53] and with gellan gum [25,55] had less swelling with lower pH. The difference in swelling affected the encapsulation efficiency and loading capacity of MB, and thus, in future studies, more pH values should be studied. The GG:Alg microparticles were influenced by pH, for example, swelling and encapsulation efficiency can be useful when applying these microparticles as DDS. The normal human body pH is 7.4. However, this value can change, for example to values around pH 6.5, like the one studied, within cancerous areas [56,57].

Regarding encapsulation efficiencies with MB, due to the deprotonation at neutral pH, there will be more anionic moieties in both polymers at pH 7.4. As MB is a cationic drug, at pH 7.4, there will be more electrostatic interactions between MB and the anionic moieties of the polysaccharides than at pH 6.5 [58]. At a more acidic pH, there is also a higher concentration of H^+^ protons that will compete with MB for the vacant anionic moieties [59]. Additionally, at neutral pH, the microparticle’s swelling is more pronounced, which helped the trapping of MB [53]. Othman et al. [33] used alginate particles for MB removal from residual waters. The adsorption capacity of the particles improved with an increase in pH. These results are in accordance with our results. Following the MB entrapment, the GG:Alg microparticles were then dried. The particles suffered no agglomeration, but did not retain the spherical form that they previously had. In this study, the encapsulation method was adsorption. It was possible to load the drug within the GG:Alg solution that was extruded; however, a significant part could be released within the CaCl_2_ bath, and MB would also contaminate the coaxial air flow system, leaving traces that could not be easily washed, requiring a more profound cleaning of the system.

In TGA, the microparticles with MB did not lose as much mass as the microparticles without the model drug. Since MB did not fully degrade at the final temperature, its presence within the microparticles reduced the mass loss. The difference in mass loss observed between particles loaded at pH 6.5 and pH 7.4 might be explained by loading capacity (Figure 5b). Higher loading capacities were obtained with pH 7.4, and thus, there was more MB within these particles. Hence, less mass was lost with microparticles loaded within PBS pH 7.4. Additionally, the difference might be due to an interaction between MB and the polymers, leading to more stable complexes. Temeepresertkij et al. [58] found that the carboxylates of alginate interacted with the N, C, and S of MB, leading to more stable alginate/MB complexes. Gellan gum also has carboxylates in its structure, and thus, there also might be a similar interaction between MB and gellan gum [48].

The microparticles had a higher mass loss with neutral pH than with pH 6.5, having a mass loss of around 50% at the end of 60 days. Alginate degrades at a faster rate with pH 7.4. Deprotonation of the carboxylic acid may lead to a faster dissolution of the alginate structure. Additionally, ionically linked alginates lose the divalent cations that form the structure and degrade at neutral pH [60,61,62]. In gellan gum, FTIR analysis made by Zhao et al. [63] confirmed that under acidic conditions (in their case pH 1.2) hydrogen ions diminished the electrostatic repulsions of the chains and thus promoting the formation of the doubled helices structure, and thus, forming more resistant structures. This is also supported by Picone et al. [64], who, with rheological studies, found that at neutral pH, the formed gellan gum gels were more fragile and deformable than the ones formed at acid mediums. Su et al. [65] studied an ionically crosslinked alginate scaffold, which was submersed in a PBS solution (pH 7.4). At the end of 25 days, 90% of the initial weight had been lost. In another study [66], 3D-printed ionically crosslinked alginate scaffolds lost half of their weight within 7 days. Regarding gellan gum, this polysaccharide is more stable in PBS. Zu et al. [67] analyzed gellan gum with different types of crosslinking. In PBS (pH 7.4) gellan gum only lost around 20% in weight at the end of 42 days. In another study, Silva-Correia et al. [68] also had similar results with the immersion of ionically crosslinked gellan gum in PBS, with also a loss of 20% at the end of 30 days. Comparing the two polymers, alginate generally degrades faster than gellan gum. The weight that was lost during the 60 days in the GG:Alg particles might be due to a more pronounced alginate degradation than gellan gum. In an artificial urine solution, Barros et al. [69] compared the degradation of alginate and gellan gum. At the end of 60 days, alginate had lost all the weight, while gellan gum maintained more than 50% of the weight. These microparticles endured within PBS solutions almost for 2 months, thus serving as possible devices for DDS. However, degradation studies need to be performed for larger times and in similar conditions to the in vivo environment. Additionally, due to size, they can be used in different areas, such as tissue engineering. These can also be used as cell carriers and serve as matrixes for cell seeding and proliferation [27,70].

The average release rate in PBS pH 6.5 was higher than with pH 7.4; however, no statistically significant differences were observed in the release profiles between the two pH. The microparticles were revealed to have a Fickian release profile, and thus, diffusion was the main driver of the release. Thus, the higher swelling indexes of the microparticles registered in pH 7.4 do not alter the release profile (in comparison with pH 6.5). The introduction of *T_lag_* to the KP and SP models improved the fittings, similar to earlier studies to be more adequate for similar particles [43,44]. In in vitro drug release studies, Jana et al. [28] had their microspheres with a good fitting with the KP model with a Fickian release mechanism. With alginate particles [71], morin was released via a Fickian diffusion profile also with the KP model. Tu et al. [72]. Prepared alginate particles via spray coagulation and then loaded with MB. A good fit with the Higuchi model was obtained, where the release was also controlled by diffusion. On the other hand, Voo et al. [73] prepared alginate particles with higher stiffness and then loaded them with MB. Unlike the previous study, the release profile from the particles was found to be non-Fickian by the KP model. In a different study [74], gellan gum beads loaded with methotrexate had a release profile with a good fit with the KP model with a Fickian release profile. Different drugs should be experimented with these microparticles to understand if the release profile remains Fickian and if the release time is similar. The release profile occurred within 6 days (144 days to reach the plateau). The release profile could be extended in future works with the addition of a protective barrier that delayed the release of the cargo, such as a hydrogel [36,75,76] or the addition of an extra layer around the microparticle, such as chitosan [77,78,79].

## 4. Materials and Methods

### 4.1. Materials

High acyl gellan gum was purchased from Sigma-Aldrich (St. Louis, MO, USA) as Phytagel. Alginic acid sodium salt was purchased from BioChemica (Panreac Química SLU, Castellar del Vallès, Spain). Methylene Blue, in powder form, was purchased from Alfa Aesar (Haverhill, MA, USA). Phosphate-buffered saline solution (PBS) with pH 7.4 was prepared following the following procedure: to 800 mL of Millipore water 4 g of sodium chloride (NaCl 100%) (J. T. Baker, Avantor, Radnor, PA, USA), 0.1 g of potassium chloride (KCl ≥ 99%) (Sigma-Aldrich, St. Louis, MO, USA.), 0.72 g of disodium hydrogen phosphate (Na_2_HPO_4_) (Panreac Química SLU, Castellar del Vallès, Spain), and 0.12 g of potassium dihydrogen phosphate (KH_2_PO_4_) (Panreac Química SLU, Spain) were added. In total, 200 mL of Millipore water was then added. For PBS with pH 6.5, the quantities were as follows: 8 g of NaCl, 0.2 g of KCl, 0.61 g of Na_2_HPO_4_, and 0.19 g of KH_2_PO_4_. Calcium chloride (Carl Roth, Karlsruhe, Germany) was used to prepare aqueous solutions of 3.5% *w*/*v*.

### 4.2. Production and Optimization of Particles Using DoE

**Figure 10:**
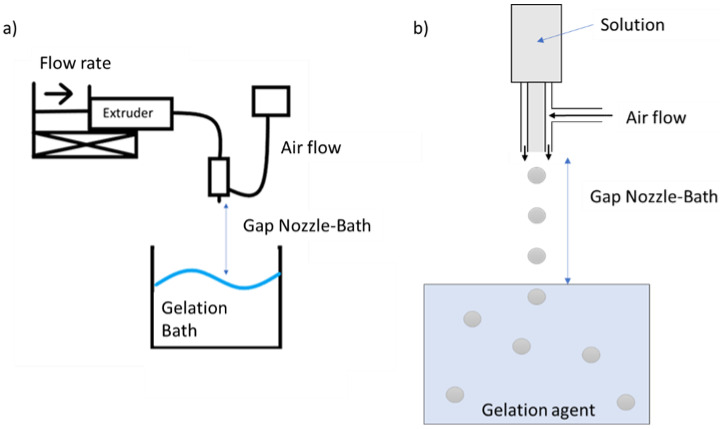
General scheme of the coaxial air flow system. (**a**) Scheme of microparticle’s production near the nozzle, adapted from from [80]. (**b**) Ampliation of (**a**).

The particles were produced using an aqueous solution (2% *w*/*v*) blend of alginate (Alg) and gellan gum (GG). A Nisco^®^ Encapsulation Unit–VARJ1 [80] was used to produce the particles via the coaxial air-flow method (Figure 10). The solution was extruded into a 500 mL calcium chloride aqueous solution (3.5% *w*/*v*). The particles were left overnight in the bath and then filtered and washed with ethanol.

To optimize the production of GG:Alg particles, a two-level fractional factorial design was used with four factors with a degree of fractionation 2 (24-1), with three replicates and a design resolution of IV. Design-Expert Software^®^ Version 11 (2018) (Stat-Ease Inc., Minneapolis, MN, USA) was used to analyze the results. Four factors were chosen: A: GG:Alg ratio within a 2% *w*/*v* aqueous solution; B: bath-nozzle gap; C: airflow; D: pump flow. The diameter (size) of the particles without drying (wet stage) was defined as a response. To evaluate dispersibility, the coefficient of variation (COV) (standard deviation/mean) and normalized SPAN (=(d(0.9) − d(0.1))/d(0.5)) were both chosen as responses. The particle size was obtained using a Leica S9 Stereo Microscope and analyzed via Image J software. At least 100 microparticle diameters were measured for each run. In DOE, a total of 24 runs of experiments were generated and executed in a randomized way. The evaluation of the responses was performed both with relevant statistical graphs (Pareto charts) and ANOVA test (confidence interval (C.I) of 95%, desired power > 90%). The significance of the mathematical model, as well as the impact of each input parameter, was determined using the Design-Expert Software^®^ 11 (2018).

The DOE had the aim of minimizing both the particle’s diameter (size) and dispersibility (analyzing SPAN and COV) using the coaxial air flow method. The DOE focused on the production of the microparticles right after their production from the coaxial airflow technique at their wet stage. After 8 h in the CaCl_2_ solution (to ensure the maximum interaction with the CaCl_2_ solution), the microparticles were filtrated and subjected to a water–ethanol exchange. For this purpose, different Millipore water and ethanol blend solutions with increasing ethanol concentration were used: (water (% *v*/*v*): ethanol (% *v*/*v*) − 90:10; 75:25; 50:50; 25:75; 10:90; 0:100). The microparticles were left for 1 h in each solution, starting from the 90:10 solution. Then, the microparticles were dried in a vacuum pump.

### 4.3. Morphological Characterization

The morphology of dried and wet particles was analyzed using an S9 Stereo Optical Microscope (Leica^®^, Wetzlar, Germany) and an SEM Tabletop microscope TM3030 Plus (Hitachi^®^, Chiyoda, Japan). Some of the optical microscopy images were also obtained in transmission mode, using an Olympus BX51 microscope (Olympus^®^, Shinjuku, Japan), coupled with an Olympus DP73 CCD camera, and acquired with the Stream Basic v.1.9 Olympus software. A cold illumination source generated by a halogen lamp (KL 2500 LCD, SCHOTT) was used. All images were obtained and automatically scaled by the respective software. In all batches of particles, at least 100 diameters were measured before analyzing.

Regarding rheological characterization of the Alg and GG solutions (2 wt%), the solutions with different ratios were analyzed in an Anton Paar MCR 502 with plate–plate geometry with 25 mm of diameter (PP25) and a 1 mm gap. Flow curves were performed at ambient temperature.

### 4.4. Swelling

Swelling in mass (*S_w_*) was calculated using Equation (1). Batches with 0.02 g of particles were submerged in 10 mL of PBS with pH 6.5 and pH 7.4. At different time intervals, batches were filtered, weighted, and analyzed to determine their diameter. Six replicas of microparticles to each pH were analyzed. Their diameter was also measured with at least 100 diameters measurements for each batch. In Equation (1), *m*_0_ is the initial mass of the particles (dried state) and *m_tw_* is the wet mass at each time:

(6) $$S_{w}=\frac{m_{tw}-m_{0}}{m_{0}}$$

### 4.5. In Vitro Degradation

The degradation was obtained by submerging particles in PBS solutions with pH 6.5 and pH 7.4, for different times within a maximum time range of 60 days (2 months). Different batches of particles were collected at different times, with six replicas to each pH. When collected, the particles were filtered, rinsed with water, and lyophilized. Then, the mass loss was determined using Equation (2), where *m_td_* is the particle’s dried mass at each time:

(7) $$mass\;loss\;(\%)=\left(\frac{m_{0}-m_{td}}{m_{0}}\right)\times 100$$

### 4.6. Encapsulation Efficiency and Loading Capacity

MB was added via adsorption, where the dried microparticles were placed within MB solutions to swell and capture the model drug. The encapsulation efficiency (EE%) and loading capacity (LC%) were determined by adding 0.02 g of particles to 5 mL of PBS with pH 6.5 and pH 7.4 with different concentrations of MB (10, 30, 60, 100, 140, 250, and 290 μg/mL). After 4 days at 37 °C on an orbital shaker, the concentration of the free MB in solution was determined using UV–VIS spectroscopy on a T90+ UV/VIS Spectrometer (PG Instruments Ltd., Lutterworth, UK) at 664 nm, with MB maximum absorbance. The calibration curves for MB concentration in PBS pH 7.4 and PBS pH 6.5 were determined to be *abs* = 0.1919*conc* + 0.0559 (R^2^ = 0.99) and *abs* = 0.1865*conc* + 0.0578 (R^2^ = 0.99), respectively (*abs* being absorbance and *conc* being concentration). EE% and LC% were determined using the following equations:

(8) $$EE\left(\%\right)=\left(\frac{{ms}_{0}-{ms}_{f}}{{ms}_{0}}\right)\times 100;LC\left(\%\right)=\frac{m_{encap.MB}}{m_{GG:Alg}}\times 100$$

where ${ms}_{0}$ is the mass of MB in the initial solution, ${ms}_{f}$ is the mass after the swelling of the particles, $m_{encap.MB}$ is the mass of encapsulated MB, and $m_{GG:Alg}$ is the weight of the particles. Four replicas were used.

### 4.7. In Vitro Fourier-Transform Infrared Spectroscopy (FTIR) and Thermogravimetric Analysis (TGA)

FTIR analysis was carried out in a Perkin-Elmer-Spectrum Two (Waltham, MA, USA). TGA analyses were performed on particles with and without MB to analyze any possible differences with the addition of MB. The analysis was carried out with a TGA-DSC-STA 449 F3 Jupiter equipment (NETZSCH, Selb, Germany). It was performed in a temperature range of 25–800 °C with a 10 K/min heating rate under a nitrogen atmosphere.

### 4.8. In Vitro Drug Release

For drug release tests, the MB concentration with the highest encapsulation efficiency was used to prepare the MB loaded particles. Batches with 0.025 g of MB loaded particles were loaded within a donor-recipient made of a permeable membrane (Spectra/Por 4 membrane, molecular cut-off 12–14 kDa). The microparticles were within the closed membranes (donor-recipient). This recipient was then submerged in 50 mL of PBS solutions with pH 6.5 and pH 7.4. The systems were kept at 37 °C with orbital agitation. At regular periods (0, 0.25, 1, 3, 6, 24, 48, 72, 144, 192, 240, and 312 h (h)), 2 mL of the PBS was retrieved and replaced with fresh PBS. The 2 mL were then analyzed by UV–VIS spectroscopy to determine the concentration of MB at each time. Five replicas were used.

## 5. Conclusions

In this study, the production method of coaxial airflow was extensively studied using a statistical approach to develop GG-based microparticles (GG:Alg) with monodisperse size. The optimization process demonstrated a direct relationship between the air flow and pump flow and particle diameter. The airflow and viscosity of the solutions were found to affect the diameter dispersibility. The particles greatly decreased their size with drying to a range of diameters from 400 to 150 μm. Due to the change of anionic moieties within GG and Alg with pH, the particles had different behaviours for environments of pH 6.5 and pH 7.4, including swelling behaviour, drug encapsulation, and release. The particles swelled more and had higher encapsulation efficiencies at neutral pH compared to acidic pH. With the modified KP and PS models, the particles showed Fickian release profiles. With this work, it was possible to understand the main factors that determine the diameter and dispersibility of GG:Alg microparticles using the coaxial air flow method. In summary, the GG:Alg particles were found to be a viable way of developing a future DDS. Future work includes the use of these microparticles in more complex systems to evaluate their potential as injectable DDS, with different drugs.

## Figures and Tables

**Table 1:** Design of experiments and responses.

RUN	FACTORS				RESPONSES		
	A: GG: Alg Ratio (%)	B: Bath-Nozzle Gap (cm)	C: Air Flow (L/min)	D: Pump Flow (mL/h)	Size (µm)	COV	SPAN
**1**	25:75	20	5	10	383.4	0.0770	0.1969
**2**	50:50	20	5	5	434.6	0.0662	0.1486
**3**	50:50	10	2.5	5	652.6	0.0659	0.1582
**4**	50:50	10	5	10	496.1	0.0400	0.1611
**5**	25:75	20	2.5	5	617.6	0.0648	0.1610
**6**	25:75	10	5	5	427.0	0.1200	0.2460
**7**	25:75	20	5	10	441.7	0.0949	0.2311
**8**	25:75	10	5	5	418.1	0.1109	0.2496
**9**	50:50	20	2.5	10	666.9	0.0619	0.1473
**10**	50:50	10	2.5	5	607.4	0.0618	0.1447
**11**	25:75	10	2.5	10	649.0	0.0600	0.1447
**12**	50:50	20	5	5	453.3	0.0690	0.1740
**13**	25:75	10	2.5	10	655.6	0.0718	0.1723
**14**	50:50	20	2.5	10	619.5	0.0653	0.1604
**15**	50:50	10	5	10	435.0	0.0589	0.1505
**16**	50:50	10	2.5	5	591.0	0.0587	0.1623
**17**	25:75	20	5	10	490.6	0.0853	0.1935
**18**	50:50	20	2.5	10	692.8	0.0735	0.1838
**19**	50:50	20	5	5	405.2	0.0825	0.1752
**20**	25:75	10	2.5	10	676.4	0.0586	0.1679
**21**	50:50	10	5	10	418.4	0.0820	0.2185
**22**	25:75	20	2.5	5	527.9	0.0796	0.1928
**23**	25:75	20	2.5	5	588.0	0.0619	0.1646
**24**	25:75	10	5	5	474.1	0.0907	0.2184

**Table 2:** Obtained ANOVA table parameters for the particles size (diameter) (*p*-value < 0.05).

Source	Sum of Squares	df	Mean Square	F-Value	*p*-Value	
**Model**	2.218 × 10^5^	2	1.109 × 10^5^	81.830	<0.0001	significant
C-Air flow	2.141 × 10^5^	1	2.141 × 10^5^	158.020	<0.0001	
D-Pump flow	7647.260	1	7647.260	5.640	0.0271	
**Residual**	28,455.610	21	1355.030			
Lack of Fit	6958.180	5	1391.640	1.040	0.4303	not significant
Pure Error	21497.430	16	1343.590			
**Cor Total**	2.502 × 10^5^	23				

**Table 3:** Obtained ANOVA table parameters for the particles size’s COV (*p*-value < 0.05).

Source	Sum of Squares	df	Mean Square	F-Value	*p*-Value	
**Model**	0.0043	3	0.0014	9.43	0.0004	significant
A-Percentage in 2%	0.0015	1	0.0015	9.93	0.0050	
C-Air flow	0.0016	1	0.0016	10.33	0.0044	
AC	0.0012	1	0.0012	8.02	0.0103	
**Residual**	0.0030	20	0.0002			
Lack of Fit	0.0010	4	0.0002	1.96	0.1499	not significant
Pure Error	0.0020	16	0.0001			
**Cor Total**	0.0073	23				

**Table 4:** Obtained ANOVA table parameters for the particles size’s SPAN (*p*-value < 0.05).

Source	Sum of Squares	df	Mean Square	F-Value	*p*-Value	
**Model**	0.0148	3	0.0049	11.81	0.0001	significant
A-Percentage in 2%	0.0052	1	0.0052	12.48	0.0021	
C-Air flow	0.0068	1	0.0068	16.19	0.0007	
AC	0.0028	1	0.0028	6.78	0.0170	
**Residual**	0.0084	20	0.0004			
Lack of Fit	0.0019	4	0.0005	1.17	0.3588	not significant
Pure Error	0.0065	16	0.0004			
**Cor Total**	0.0232	23				

**Table 5:** ANOVA parameters (*p*-value < 0.05) comparing the release profiles of GG:Alg microparticles in PBS with pH 7.4 and 6.5. Statistically significant times are indicated with an *.

Time (h)	ANOVA Parameters between pH 6.5 and 7.4
24	F(1,8) = 0.311, *p* = 0.592
48	F(1,8) = 3.131, *p* = 0.115
72	F(1,8) = 0.797, *p* = 0.398
144	F(1,8) = 11.673, *p* = 0.009 *
192	F(1,8) = 17.586, *p* = 0.003 *
240	F(1,8) = 0.405, *p* = 0.542
312	F(1,8) = 0.658, *p* = 0.441

**Table 6:** Parameter values and R^2^_adj_ from the fittings of the mathematical models from the release profiles, from DDSolver. The best fit for each system is in bold.

pH		pH 6.5	pH 7.4
KP	*k*	20.760	19.675
	*n*	0.239	0.238
	*R* ^2^ *_adj_*	**0.8313**	**0.8461**
KP *T_lag_*	*k*	67.111	50.531
	*n*	0.044	0.081
	*T_lag_*	23.994	14.713
	*R* ^2^ *_adj_*	**0.9915**	**0.9819**
Wbll	*a*	10.989	8.095
	*b*	0.591	0.466
	*R* ^2^ *_adj_*	**0.9279**	**0.9201**
PS	*k*_1_	12.637	11.920
	*k*_2_	−0.428	−0.405
	*m*	0.446	0.445
	*R* ^2^ *_adj_*	**0.9228**	**0.9365**
PS *T_lag_*	*k*_1_	35.742	31.730
	*k*_2_	−3.575	−2.991
	*m*	0.264	0.273
	*T_lag_*	5.999	5.994
	*R* ^2^ *_adj_*	**0.9893**	**0.9914**

## Data Availability

Data is available within article.
